# Radiotherapy for early glottic cancer and salvage surgery after recurrence

**DOI:** 10.1590/S1808-86942011000300005

**Published:** 2015-10-19

**Authors:** Paulo Pontes, Osíris de Oliveira Camponês do Brasil, Francisco de Souza Amorim Filho, Bruno Teixeira de Moraes, Antonio Pontes, José Caporrino Neto

**Affiliations:** 1 Full Professor - Department of Otorhinolaryngology and Head and Neck Surgery of the Federal University of São Paulo, UNIFESP-EPM; 2 PhD in Sciences; Professor - Department of Otorhinolaryngology and Head and Neck Surgery of the Federal University of São Paulo, UNIFESP-EPM; 3 Head and Neck Surgeon; PhD in Sciences - Department of Otorhinolaryngology and Head and Neck Surgery of the Federal University of São Paulo, UNIFESP-EPM; 4 Otorhinolaryngologist; MSc student - Department of Otorhinolaryngology and Head and Neck Surgery of the Federal University of São Paulo, UNIFESP-EPM; 5 Otorhinolaryngologist; MSc student - Department of Otorhinolaryngology and Head and Neck Surgery of the Federal University of São Paulo, UNIFESP-EPM; 6 PhD in Sciences; Assistant Physician - Department of Otorhinolaryngology and Head and Neck Surgery of the Federal University of São Paulo, UNIFESP-EPM; Institute of the Larynx (INLAR)

**Keywords:** laryngeal neoplasms, laryngectomy, neoplasm recurrence local, radiotherapy, salvage therapy

## Abstract

Early glottic cancer can be effectively treated with radiation or surgery, but recurrence is a possibility when using any of the treatment modalities.

**Aim:**

To assess the outcome of radiotherapy as initial treatment in the control of squamous cell carcinoma of vocal cord (T1) and the effectiveness of salvage surgery (endoscopic or open) after treatment failure.

**Materials and Methods:**

A retrospective study was based on the analysis of medical records from 43 patients with T1 squamous cell carcinoma of the glottis, radiotherapy as initial treatment and follow-up period of five years.

**Results:**

The rate of recurrence after radiotherapy was 30.2% of the cases, mean diagnosis interval was 29.5 months. As an option for salvage treatment, patients underwent open partial laryngectomy or endoscopic surgery with control rates of 77.7% and 25% respectively.

**Conclusion:**

Our cases showed high rates of recurrence after radiotherapy and open partial laryngectomy was more effective for the salvage surgery.

## INTRODUCTION

Malignant laryngeal neoplasm is one of the most frequent diseases of the head and neck, and in 90% of the cases it is represented by the epidermoid carcinoma and its genesis is directly associated with alcohol drinking and smoking.

It more frequently affects the glottis - 64% of the cases, and any epithelial disarray or change caused by the tumor will change the vibratory movements of the vocal folds, causing dysphonia. Of all the glottis tumors, 56% are diagnosed in their initial stages (T1)^1^.

The primary glottis cancer can be effectively treated with radiotherapy or surgery (endoscopic or open) and it bears some of the best cure rates among the malignant neoplasms of the aerodigestive tract. In the series published by Davis et al.^2^, they reported a local control rate of 93% in patients with glottic T1 tumors submitted to surgery and 67% in T1 patients submitted to radiotherapy. Nonetheless, when we consider only the T1a stage tumors, some authors report similar control rates with cordectomy and radiotherapy, 84% and 77% respectively.^3^

Treatment must not be restricted only to curing the cancer, which is the main objective, but one must also attempt to preserve breathing, swallowing and phonation.

## OBJECTIVE

To assess the results of radiotherapy as the initial treatment mode in controlling the epidermoid carcinoma of the vocal fold (T1) and the efficacy of surgical salvage (endoscopic or open) after initial treatment failure.

## MATERIALS AND METHODS

The present study was approved by the Ethics in Research Committee of the institution to which this study is associated, under registration number 0353/10.

In this series we retrospectively assessed the charts from 43 patients with T1 stage glottis epidermoid carcinoma (42 men and 1 woman), submitted to radiotherapy as initial treatment, who were first seen by a doctor between 1990 and 2002.

All the patients were sorted according to the TNM-UICC 2002 classification, after indirect laryngoscopy obtained by tele-laryngoscopy and/or nasal fibroscopic laryngoscopy (saved in video) and diagnostic confirmation of the epidermoid carcinoma through biopsy/frozen section.

We took off the sample those patients who had been previously treated in other services, those with tumors bearing other clinical stages and those with tumors of histological lineage other than the epidermoid carcinoma.

The routine in our service is to present all treatment options (radiotherapy or surgery) to patients with T1 glottic carcinoma, explaining the respective pros and cons; nonetheless, surgery is our treatment of choice for these cases. Radiation treatment was based on external beam at the dose of 6,000 to 6,800 rad in 30–34 fractions, indicated according to two basic criteria:

• Patient clinically ineligible for surgery (n=12/28%)
• Patient's wish (n=31/72%)

In order to analyze the results obtained by radiotherapy, the patients were grouped according to primary tumor localization on the vocal folds as follows:

• Group A - T1a: Tumor not involving the anterior commissure (n=22).
• Group B - T1b: Tumor involving the anterior commissure with or without invasion of the contralateral fold anterior third (n=21).

After radiotherapy, all the patients were followed up for a period of five years or more, and in the first six months they returned monthly to the clinic. For clinical control we did indirect laryngoscopy with the goal of identifying any suspicious lesions early on. After tumor recurrence diagnosis, the situation was recorded in the patient's chart and the patient was referred to salvage treatment as soon as possible, which first option was partial open laryngectomy (frontolateral vertical) in the cases with or without commissure involvement. Patients with T1a tumors upon recurrence who were clinically unfit were submitted to an endoscopic tumor resection (transmuscular cordectomy), since it is an approach with less morbidity than open surgery.

## RESULTS

The patients submitted to radiotherapy (N=43) to treat initial glottic cancer had local recurrence in 30.2% (N=13) of the cases. Everyone who had recurrences was male with an average age of 58.9 years. The mean time between radiotherapy onset and local recurrence diagnosis was 29.5 months. The most frequent histology type was the moderately differentiated epidermoid carcinoma (76.9%), followed by the well differentiated type (23.1%).

As to primary tumor location, initial radiotherapy treatment had the following recurrences: In Group A, where the initial lesion did not involve the anterior commissure, there was recurrence in 31.8% (n=7) of the cases (Table 1); in Group B, in which the initial lesion invaded the anterior commissure with or without involvement of the contralateral vocal fold, the recurrence rate was 28.5% (n=6), slightly lower than that of the previous group, when radiotherapy was carried out (Table 2).

**Table 1:** Group A (T1a): treatment by radiotherapy versus tumor recurrence.

Recurrence	Radiotherapy	
	N	%
With recurrence	7	31,8
Without recurrence	15	68,2
Total	22	100

**Table 2:** Group B (T1b): treatment by radiotherapy versus tumor recurrence.

Recurrence	Radiotherapy	
	N	%
With recurrence	6	28,5
Without recurrence	15	71,5
Total	21	100

As soon as the failure in radiotherapy was confirmed, the patients were once again staged, being similar to the staging before radiotherapy with no loco regional metastasis. Four patients were referred to salvage endoscopic surgery (transmuscular cordectomy), because they were clinically unfit. Nine patients were submitted to open surgery (vertical-frontolateral laryngectomy) without neck dissection (Table 3).

**Table 3:** Local recurrence according to treatment.

Recurrence	Treatment		Total
	Open surgery	Endoscopic procedure	
Cured	77,7% (n=7)	25% (n = 1)	69,20%
Uncured	22,2% (n=2)	75% (n=3)	30,80%
Total	N = 9	N=4	N=13

Of the recurrence cases treated endoscopically (n=4), only one was cured, the remaining patients (n=3) were referred to total laryngectomy because of a new recurrence. In this group, one patient died of cancer.

In the cases submitted to open surgery, the cure rate was higher, and 77.7% (n=7) patients benefited from surgery. 22.2% (n=2) of the patients had new treatment failure and were referred to total laryngectomy. In this group we also had one patient dying of cancer.

## DISCUSSION

The treatment of laryngeal cancer must always associate oncologic efficiency with function preservation. Traditionally, initial glottic carcinomas (T1) can be effectively treated through surgery or radiotherapy, with high local control and survival rates^4^, ^5^. Despite radiotherapy's complications such as persistent edema, glottic stenosis or hypothyroidism, its advantage is that phonation and swallowing disorders are usually less impacting that the surgical procedures^6^, ^7^. According to Brasil et al.^8^, ^9^, while radiotherapy tends to keep the glottic region as the sound source, in patients submitted to surgery such source tends to shift to the supraglottic region, with a worsening in vocal quality, but keeping a socially acceptable voice, especially when there is speech therapy associated.

According to Bron et al.^3^, T1a carcinomas had similar local control in 5 years with radiotherapy or cordectomy, 77% and 84% respectively. In T1b carcinomas, radiotherapy had lower success rates when compared to surgery (supra-cricoid partial laryngectomy), 66% and 100% respectively. Other papers report 80 to 95%^10^, ^11^ success rate of radiotherapy in T1 laryngeal carcinomas. Our results with radiotherapy to treat T1a and T1b tumors showed high rates of recurrence. Therefore, we believe that surgical treatment is the best option to control this disease, especially in T1b cases with anterior commissure invasion suspicion.

There is a current trend towards performing more conservative surgical procedures in cases of tumor recurrence after radiotherapy, instead of doing a total laryngectomy. In a study carried out by Schwaab et al.^12^, patients with initial glottic tumors (T1a, T1b and T2 with preserved vocal cord mobility) who had recurrence after radiotherapy were submitted to total or partial laryngectomy (vertical or subtotal with crico-hyoid-pexia) with 77% and 100% loco regional control in 5 years, respectively. According to Ganly et al.^13^, patients with recurrence after radiotherapy for initial tumors, treated by total or partial laryngectomy, had global survival rates of 50% and 89%, respectively, with similar rates of complications, showing that there can be good disease control and preservation of laryngeal functions with partial surgery, when feasible^14^.

Among partial laryngectomies, the endoscopic approach in cases of post-radiotherapy recurrence must be avoided, having in mind the low rate of loco regional disease control, besides missing on an opportunity to do an open surgery with partial laryngeal preservation, as shown in the present study. This is explained by the difficulty in establishing lesion margins in an accurately way in a previously irradiated larynx, associated with the limitation of achieving broad margins.

## CONCLUSION

In our cases, initial glottic tumors (T1) treated by radiotherapy had high rates of recurrence. This can be a treatment option, but the patient must be aware that higher cure rates can be achieved through surgery.

Open partial laryngectomy without neck dissection is an efficient option to treat glottic cancer after radiotherapy failure in patients who remain in stages T1a or T1b, bearing satisfactory cancer control without the mutilation of a total laryngectomy.
